# Atherosclerotic Risk Factors in Children with Celiac Disease

**DOI:** 10.1155/2020/6138243

**Published:** 2020-03-27

**Authors:** Anna Rybak, Aldona Wierzbicka, Piotr Socha, Anna Stolarczyk, Bożena Cukrowska, Łukasz Obrycki, Zbigniew Wawer, Roman Janas, Beata Oralewska, Anna Szaflarska-Popławska, Barbara Iwańczak, Elżbieta Cyrta-Jarocka, Urszula Grzybowska-Chlebowczyk, Wojciech Cichy, Grażyna Czaja-Bulsa, Jerzy Socha

**Affiliations:** ^1^Department of Gastroenterology, The Great Ormond Street Hospital, London, UK; ^2^Department of Biochemistry and Experimental Medicine, Children's Memorial Health Institute, Warsaw, Poland; ^3^Department of Gastroenterology, Hepatology and Feeding Disorders, Children's Memorial Health Institute, Warsaw, Poland; ^4^Department of Pathology, Children's Memorial Health Institute, Warsaw, Poland; ^5^Department of Nephrology, Kidney Transplantation and Arterial Hypertension, The Children's Memorial Health Institute, Warsaw, Poland; ^6^Department of Pediatrics, Allergology and Gastroenterology, Collegium Medicum Nicolaus Copernicus University, Bydgoszcz, Poland; ^7^Department of Pediatrics, Gastroenterology and Nutrition, Medical University of Wroclaw, Wroclaw, Poland; ^8^Department of Pediatrics, Gastroenterology and Allergology, Medical University, Białystok, Poland; ^9^Department of Gastroenterology, Department of Pediatrics, Upper-Silesia Center of Children's Health, Katowice, Poland; ^10^Department of Pediatrics, Gastroenterology and Metabolic Diseases, University of Medical Sciences, Poznan, Poland; ^11^Department of Pediatrics, Gastroenterology and Rheumatology, Independent Specialist Public Health Care, Szczecin, Poland

## Abstract

**Results:**

We found significantly lower concentrations of total cholesterol, lipoprotein LDL-C, apolipoproteins A1 and B, as well as hCRP in all children with CD. We showed decreased level (<5 ng/mL) of folic acid among 46% of children treated for >5 years. Moreover, we showed significant decrease of folic acid level already after 1 year of a GFD (12 *vs*. 5.6 ng/mL; *p* < 0.001). We also found significant negative correlation of *z*-score body mass index (BMI) with HDL and APOA1 level (*r* = −0.33; *p* = 0.015 and *r* = −0.28; *p* = 0.038, respectively) and modest positive correlation of *z*-score BMI with atherogenic factor of total cholesterol-HDL ratio and LDL-HDL ratio (*r* = 0.40; *p* = 0.002 and *r* = 0.36; *p* = 0.006, respectively). Analysis of physical activity showed an increase in the insulin levels with inactivity (*r* = 0.36; *p* = 0.0025). We also found positive correlation of the sleep duration with the adiponectin level (*r* = 0.41; *p* = 0.011).

**Conclusions:**

In children with CD treated with a GFD, decreased level of folic acid together with increased BMI, sedentary behavior, and an improper lipid profile may predispose them to atherosclerosis in the long run. This data suggests the need of further studies to determine the need for metabolic cardiovascular risk screening in children with CD.

## 1. Introduction

Celiac disease (CD) is a complex autoimmune disorder occurring in genetically susceptible individuals, with the prevalence of 0.3%–2% in the Western populations [1, 2], characterized by intolerance to gluten peptides. CD results in a combination of different, gluten-dependent symptoms, enteropathy, and the appearance of specific auto-antibodies in HLA-DQ2 or DQ8-positive individuals. CD is a chronic condition, requiring lifelong treatment with a gluten-free diet (GFD). Untreated CD causes inflammatory processes and often impaired nutritional status; however, lifelong treatment with a GFD and therefore possible nutritional impairments may impact long-term health and concomitant diseases. Some studies showed that dietary interventions in childhood may impact physical condition later in life. There is limited and ambiguous data on the impact of a GFD on the risk of developing cardiovascular diseases [3–6]. As cardiovascular disease appears to be an inflammatory process leading to endothelial damage [7], several factors are additionally regarded to describe cardiovascular risk, including proinflammatory cytokines and C-reactive protein (CRP). Adipocytokine has been claimed to be a good marker of the metabolic syndrome in children [8]. Sedentary behavior is an independent factor which should be taken into account, and as previously shown, physical activity can improve arterial function [9]. Additionally, Raitakari et al. studied the tracking of physical activity and its influence on selected coronary heart disease risk factors in a 6-year study of Finnish adolescents and young adults, and they showed that subjects who are constantly inactive express a less beneficial coronary risk profile compared with those who are constantly active [10].

Taking reports concerning increased cardiovascular episodes among patients with CD into consideration, we present hereby our study concerning the impact of a one-year treatment with a GFD, as well as level of physical activity, on the lipid profile and biochemical risk factors of atherosclerosis in children with celiac disease.

## 2. Material and Methods

**Figure 1:**
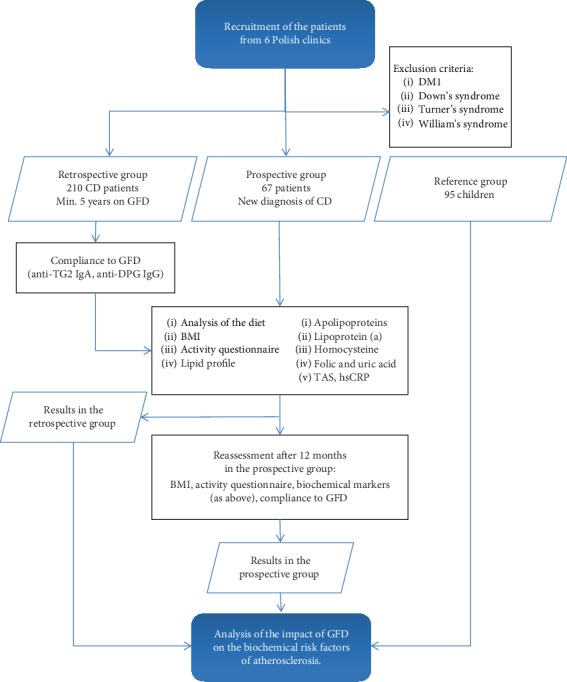
Flowchart of the project plan. CD: coeliac disease; GFD: gluten-free diet; anti-TG2: tissue transglutaminase antibody; anti-DPG: deamidated gliadin peptide antibodies; BMI: body mass index; TAS: total antioxidant status; hsCRP: high-sensitivity C-reactive protein.

We enrolled 277 patients with CD from 7 Polish clinics (Warsaw, Bydgoszcz, Poznan, Wroclaw, Bialystok, Katowice, and Szczecin) into the study. 210 children diagnosed and treated for CD for at least 5 years were recruited into the retrospective group. 67 children with newly diagnosed CD were recruited to the prospective group and observed for 1 year after introduction of the GFD. The exclusion criteria included cooccurrence of type 1 diabetes mellitus, Down's, William's, and Turner's syndromes and applied to both groups, including one year of observation in the prospective group. The project's plan is shown on the flowchart in Figure 1.

The CD diagnosis was established in particular centers according to the European Society for Paediatric Gastroenterology Hepatology and Nutrition (ESPGHAN) criteria, applicable for a given time interval [11]. For each patient, extended clinical and anthropometric data (weight, height, and body mass index (BMI)) were obtained using a unified questionnaire completed by pediatrician or pediatric gastroenterologist in each center. Food and activity questionnaire was completed together with caregivers and/or patients. From each patient, a blood sample of 5 mL (to clot) and 2.7 mL (EDTA) were collected for both, specific antibodies and biochemical markers assessment. Using anti-tissue transglutaminase antibodies (anti-TG2 IgA) and anti-deamidated gliadin peptide antibodies (anti-DGP IgG), all patients in the retrospective group and patients in the prospective group after 12 months from the diagnosis were evaluated for the compliance to the GFD. The tests were performed using immunoenzymatic assays from Celikey-Varelisa (Phadia) and GAF3x (Euroimmun) according to manufacturer's recommendations. The positive result was recognized with anti-TG2 titer of more than 8 UI/mL and anti-DPG titer over 25 UI/mL.

The lipid profile (total cholesterol (TCh), triglycerides (Tg), low-density cholesterol (LDL-C), very low-density cholesterol (VLDL-C), and high-density cholesterol (HDL-C)), atherosclerotic indicators (TCh/HDL-C and LDL-C/HDL-C), apolipoproteins (APOA1, B, and E), lipoprotein (a), homocysteine, antioxidants (folic and uric acid), total antioxidant status (TAS), and high-sensitivity CRP (hsCRP) were assessed from the blood samples—once in the retrospective group and twice in the prospective group (on the day of diagnosis and 12 months post the GFD introduction).

As a reference group, we used the data of 95 healthy children (all 8 years old) recruited in another project, for which we previously had the results of selected biochemical parameters (lipid profile, apolipoprotein A1, B, atherogenic factors, and hsCRP).

Cholesterol was measured by spectrophotometry (available on request, detection limit: 4.3 mg/dL = 0.11 mmol/L, linearity limit: 1000 mg/dL = 26 mmol, and repeatability (within run): 121 mg/dL = 3.13 mmol/L inter- and intra-assay CV 1.1%; BioSystems S.A., Barcelona, Spain). Triglycerides were determined by a spectrophotometric method based on coupled reactions with lipase, glycerol kinase, and G-3-P oxidase (triglyceride kits available on request, detection limit: 4.4 mg/dL = 0.05 mmol/L, linearity limit: 600 mg/dL = 6.78 mmol/L, and repeatability (within run): 44 mg/dL = 0.50 mmol/L inter- and intra-assay CV 2.8%; BioSystems S.A., Barcelona, Spain).

Levels of the HDL cholesterol were determined spectrophotometrically (BioSystems S.A., Barcelona, Spain). Cholesterol from low-density lipoproteins (LDL), very low-density lipoproteins (VLDL), and chylomicrons was measured by an enzymatic accelerated noncolor forming reaction. In order to determine the low-density lipoproteins (LDL) in the sample, it was precipitated with polyvinyl sulphate. The cholesterol concentration was calculated from the difference between the serum total cholesterol and the cholesterol in the supernatant after centrifugation. Lipoproteins of very low-density (VLDL) were obtained by density gradient ultracentrifugation with NaCl and then cholesterol was measured in the isolated fraction (cholesterol HDL kit, available on request, detection limit: 0.5 mg/dL = 0.01 mmol/L, linearity limit: 200 mg/dL = 5.18 mmol, and repeatability (within run): 32.9 mg/dL = 0.85 mmol/L inter- and intra-assay CV 1.3%; cholesterol LDL kits, available on request, detection limit: 0.45 mg/dL = 0.01 mmol/L, linearity limit: 1000 mg/dL = 26 mmol, and repeatability (within run): 120 mg/dL = 3.11 mmol/L inter- and intra-assay CV 2.8%; and VLDL cholesterol detection limit: 0.45 mg/dL = 0.01 mmol/L, linearity limit: 1000 mg/dL = 26 mmol, and repeatability (within run): 150 mg/dL = 3.94 mmol/L inter- and intra-assay CV 4.5%).

apolipoprotein A1 (ApoA1) and apolipoprotein (AB) were measured by a liquid-phase immunoprecipitation assay with nephelometric endpoint detection (ApoA1 and ApoB detection limit: 0.06 g/L, linearity limit: 3.50 g/L, and repeatability (within run): 10 g/L, inter- and intra-assay CV 4.7%; Orion Diagnostica, Finland).

For lipoprotein (a) determination, we used an assay from Sebia which is based on an electroimmunodiffusion technique. In this Laurel rocket method, monospecific anti-Apo (a) antibody is incorporated into an agarose gel with quantitation determined by comparing sample rocket heights against a standard curve.

We measured homocysteine with the architect homocysteine assay which is based on chemiluminescent assay (CMIA) technology, with flexible assay protocols (Abbott Park, USA).

For the determination of total antioxidant status (TAS), we used ABTS® (2, 2′-Azino-di-[3-ethylbenzthiazoline sulphonate]) which was incubated with a peroxidase (metmyoglobin) and H_2_O_2_ to produce the radical cation ABTS®∗+. This has a relatively stable blue-green color, which is measured at 600 nm. Antioxidants in the added sample cause suppression of this color production to a degree which is proportional to their concentration Randox total antioxidant status (Randox Laboratories Ltd., Ardmore, United Kingdom).

For high-sensitivity CRP (hsCRP), ELISA kits (sensitivity: 0.54-18 mg/L; inter- and intra-assay CV: 4.2 were from the DRG International, Inc., Springfield, USA) were used.

Uric acid was measured by spectrophotometry (uric acid kits, available on request, detection limit: 0.2 mg/dL = 25 mg/dL and linearity limit: 25 mg/dL = 1487 *μ*mol/L, inter- and intra-assay CV 2.1%; BioSystems S.A., Barcelona, Spain).

Activity questionnaires were obtained from all patients and it comprised three subgroups of physical activity: activity (number of hours spent on sports per day), inactivity (number of hours spent in front of the TV or computer), and sleep duration (hours per day).

The informed consent was obtained from each participant and signed by a caregiver and/or patient. The project received a positive opinion from the local bioethical committee (document 71/KBE/2010).

Statistical analysis for uneven or not numerous groups which have nonnormal distribution according to Shapiro-Wilk test for normality was assessed using Mann–Whitney *U*-test with *p* value less than 0.05 considered as significant. Otherwise, Pearson *χ*^2^ test was performed. Statistical analysis was performed in SPSS (v. 15.0.1 SPSS Inc., Chicago, IL, USA) and R Statistics [12].

## 3. Results

**Figure 2:**
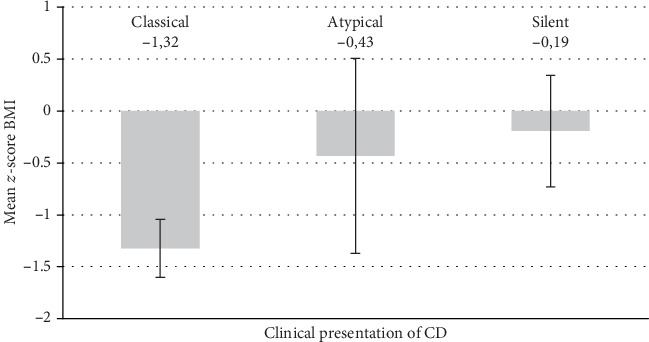
Variance analysis of the correlation between *z*-score BMI and clinical presentation of CD.

The clinical characteristics of patients enrolled into the study are shown in Table 1. We enrolled 277 patients with celiac disease, with mean age of 12.7 years (min. 18 months, max. 22 years). More than half of recruited patients presented with classical symptoms of CD, including gastrointestinal symptoms and/or failure to thrive (*n* = 156; 62%), 33% and 4.8% of patients suffered from atypical (with extra intestinal symptoms) or silent, asymptomatic CD, respectively. In 5 patients, from the retrospective group, we found positive autoantibodies, but a duodenal biopsy showed Marsh I changes. However, due to the improvement of symptoms on a GFD, these patients were included in the study. 20% of patients had concomitant diseases, with anxiety disorders and selective IgA deficiency to be the most common (4.6% and 7.2%, respectively). Slightly over a half of patients (57%) had a BMI within the normal range for age (i.e., between 10^th^ and 90^th^ centile), 8% of patients were overweight or obese (BMI > 90^th^ centile). We found no correlation between mean *z*-score BMI and clinical presentation of CD (coefficient *F* = 0.9874, *p* = 0.375, Figure 2).

The autoantibody measurements were available for 257 patients (8 blood samples were missing, and 12 results were ambiguous; therefore, these 20 patients were withdrawn from the further analysis). 75.5% of patients (*n* = 194) had a negative result for anti-TG2 IgA or anti-DGP IgG (in patients with selective IgA deficiency), therefore compliant to GFD. We found 63 patients (24.5%) with positive test for anti-TG2 or anti-DGP and therefore noncompliant to GFD.  Table 2 shows data regarding IgA anti-TG2 results in both, retrospective group (after 5 years of a GFD) and prospective group (after 1 year of GFD). We found no correlation between mean *z*-score BMI and mean increase in *z*-score BMI (in the prospective group) with compliance to the GFD (Table 3).

### 3.1. All CD Patients *vs*. Reference Group

We found significantly lower concentrations of total cholesterol, lipoprotein LDL-C, and apolipoprotein A1 and B and significantly higher level of triglycerides in all children with CD (*n* = 277) in comparison to the healthy controls (*n* = 95). Apart from the atherosclerotic indicators of APOA1/APOB, we were unable to show significant differences among other indicators like TCh/HDL-C or LDL-C/HDL-C. Summary of these results is shown in Table 4.

### 3.2. Analysis in the Prospective Group

In the prospective group (*n* = 67), the measurements were taken on the day of diagnosis and compared to the results taken 12 months after introducing GFD. There was a significant increase in the mean value of the total antioxidant status (TAS) from 1.30 mmol/L to 1.44 mmol/L (*p* = 0.039; Table 5); however, it remained within the normal range (1.30-1.77 mmol/L). We also showed a substantial decrease in hsCRP level (from 0.57 to 0.19; *p* = 0.029), as well. We found no correlation of *z*-score BMI with biochemical factors. We also found no significant differences in the levels of biochemical factors between patients compliant and noncompliant to a GFD.

At the time of diagnosis of CD, 13 children had elevated levels of total cholesterol (TCh) above 190 mg/dL. The increase in LDL-C was observed in 10 children. In 7 children, there was an increase in VLDL-C. The decrease in HDL-C, below 40 mg/dL, was found in 16 children. 14 children showed a decrease in apolipoprotein A1. The increased concentrations of lipoprotein (a) and hsCRP were observed in 8 children, and an increase in uric acid and homocysteine in 7 children. The decrease in TAS was found in 16 children, and in 15 children, decrease in the concentration of folic acid was observed. When the dietary treatment, based on the exclusion of gluten products, was introduced, there was no statistically significant difference for the tested parameters except the decrease in folic acid concentration (from 13.0 mg/L to 5 mg/L) and hsCRP (from 0.58 mg/dL to 0.39 mg/dL). In the subgroup analysis (classical and atypical CD), there was significant decrease in hsCRP (from 1.2 mg/dL and 0.6 mg/dL to 1.7 mg/dL and 0.77 mg/dL, respectively).

Physical activity was positively correlated with insulin change (at the time of diagnosis vs. 12 months on GFD). We found that physical activity was related to lower insulin levels (0.278 vs. 0.358 in active vs. nonactive patients, *p* = 0.025, correlation coefficient *r* = 0.36).

Low activity in children was positively correlated with higher homocysteine level (correlation coefficient *r* = 0.47; *p* = 0.001). There was no correlation found between physical activity and adiponectin or leptin in 12 months of observation.

There was, however, a positive correlation of the sleep duration with the adiponectin level (*r* = 0.41; *p* = 0.011) and IgF-BPr (correlation coefficient *r* = 0.35; *p* = 0.040). There was no correlation of the physical activity, inactivity, or sleep duration with *z*-score BMI, which is most probably due to the short observation time.

### 3.3. Analysis in the Retrospective Group

We found significant negative correlation of *z*-score BMI with HDL and APOA1 level (*r* = −0.33; *p* = 0.015 and *r* = −0.28; *p* = 0.038, respectively) and a modest positive correlation of *z*-score BMI with atherogenic factor of total cholesterol/HDL-C ratio and LDL-C/HDL-C ratio (*r* = 0.40; *p* = 0.002 and *r* = 0.36; *p* = 0.006, respectively) in the retrospective group (*n* = 210). We found no significant differences in total cholesterol level in all patients when compliance to the GFD was a differential variable.

In this group, there was an increase in total cholesterol in 17 children, elevated levels of lipoprotein LDL-C in 12 children, and TG increase in 5 children. In 22 children, increased concentrations of lipoprotein (a) and homocysteine were observed. The reduced concentrations of the lipoprotein HDL-C and apolipoprotein A1 were found in 32 children in the retrospective group. As many as 89 children (42.4%) had reduced levels of folic acid.

With consistency to results for all CD patients, there were significantly lower concentrations of total cholesterol, lipoprotein LDL-C, and apolipoprotein A1 and B and significantly higher level of triglycerides in the retrospective group (*n* = 210) in comparison to the healthy controls (*n* = 95) (Table 6).

There was no correlation found between compliance to the diet and mean BMI *z*-score (*p* = 0.6224) or mean increase of the BMI *z*-score (12 months post primary measurements) (*p* = 0.8409).

## 4. Discussion

In the presented study, major cardiovascular risk factors in children with celiac disease were analyzed and showed a decrease of some risk factors like cholesterol concentration and LDL-cholesterol concentration. GFD seems to decrease cardiovascular risk as indicated by lowering hsCRP and increasing antioxidant capacity, but there are some additional nutritional risks, like folate deficiency, which have to be taken into account.

There are limited studies on the impact of GFD on the risk factors of cardiovascular disease. Moreover, the data on the lack of the adherence to the GFD can significantly carry weight to the results and lead to wrong conclusions. Presented data suggest no significant changes in lipid profile after one year of GFD in children with CD when compared to the baseline results at the time of diagnosis (Table 5). The presented results show a more favorable lipid profile in all CD patients in comparison to healthy controls (Table 4), and this data seem to be consistent with previous reports. Serum level of total cholesterol is low, however with mean level within the normal range in both diagnosed and undiagnosed CD patients [[4, 9, 13, 14]. This might be due to the malabsorpion, reduced cholesterogenesis, increased biliary secretion, or high fecal elimination of cholesterol in untreated patients [15]. Lewis et al. [16] showed that total cholesterol is not only lower in CD patients compared to healthy controls but also it does not increase after following a GFD for one year. These authors observed also an increase in HDL-cholesterol in CD patients following the treatment. Brar et al. studied 132 CD patients strictly adherent to GFD for a median of 20.5 months and showed an increase in total cholesterol, as well as high-density lipoprotein (HDL), but not in low-density lipoprotein (LDL) [17]. These studies suggested that treatment with a GFD led to the improvement of the imbalanced lipid profile of the newly diagnosed or undiagnosed CD patients; thus, proper treatment and compliance to GFD would be an antiatherosclerotic factor in CD patients. The presented results did not confirm these findings in the prospective group which can be explained by favorable lipid profile on the day of diagnosis probably due to the low age in our group of patients.

On the other hand, treatment with GFD involves the risk of fiber, iron, folate, and calcium deficiency [18, 19]. As shown, there is a substantial decrease in serum folic acid level after 12 months of GFD (from 12.1 mg/L to 5.6 mg/L; *p* = 0.001; Table 5). The lowering of folate can result in elevated mean plasma homocysteine concentration and thus lead to atherosclerosis. However, low level of folate in CD patients is conflicting with the folate content in food products—cereal or whole-grain products contain less folate than fresh fruits, vegetables, or soy, which are not restricted in GFD. As previously showed, studied group of CD patients had atherogenic profile of the dietary habits including low intake of products rich in dietary fiber, natural antioxidants including fruits and vegetables, vitamins, and high intake of sweets, meats, and saturated fats [20]. Similarly, Hallert et al. showed poor vitamin status in half of CD patients, particularly plasma folate, but also pyridoxal 5′-phosphate (active form of vitamin B6) and vitamin B12 [21]. On the other hand, some countries fortify wheat flour with folate; therefore, the sudden change in bread consumption due to introducing GFD may result with rapid lowering of serum folate concentration, therefore starting GFD should be followed with balanced folate ingestion or supplementation [22]. Some countries (not yet Poland) overcame this problem by fortifying gluten-free products with folate [23], and presented data support this practical solution to avoid folate deficiency.

As CD is a chronic immune-mediated inflammatory intestinal disorder with an autoimmune component, it is important to remember that inflammation plays a key role in pathogenesis of atherosclerosis, and proinflammatory immune cells activated in CD could affect the development of atherosclerotic lesions. One of the gliadin peptides, P31–43, was shown to accumulate in lysosomes and thus increase level of the free radicals [24, 25]. Based on these findings, the increased risk of atherosclerosis in untreated CD patients could be explained by the gliadin peptide contribution in the oxidative modification of LDL. The presented data showed a significant increase of the mean value of the total antioxidant status (TAS) from 1.30 mmol/L to 1.44 mmol/L, together with a significant decrease in the inflammatory marker hsCRP, already after 12 months of CD treatment with a GFD (Table 5). These findings suggest the protective impact of a GFD on the potential risk factors of atherosclerosis. CRP high sensitivity has been proven in many studies to be a good indicator of cardiovascular risk and seems to be relevant to the clinical situation where inflammatory processes can result from gluten hypersensitivity [26].

In our study, we found higher insulin levels in patients with low physical activity (*r* = 0.36; *p* = 0.0025). This is consistent with data from the literature, suggesting significant dose-related effects between physical activity and the atherosclerosis risk factors, including insulin level [27].

### 4.1. Limitation of the Study

The study has some limitations. We did not measure atherosclerotic vascular changes directly which could be assessed by measurement of carotid artery intima-media thickness (c-IMT). De Marchi et al. showed a significant increase of c-IMT in the CD group compared to the controls [28]. They also showed that the introduction of GFD for 6–8 months resulted in a significant decrease of c-IMT in CD patients.

We found quite high number (although consistent with the literature) of CD patients noncompliant to GFD. Even though we failed to prove the impact of GFD noncompliance on the biochemical markers (both in prospective and retrospective group), our analyses were limited due to the small sample size.

The project had two arms—prospective and retrospective. Due to the latter, clinical data for some patients was incomplete (clinical presentation of celiac disease, Marsh classification; *n* = 32).

### 4.2. Strengths of the Study

The strength of our study is a wide assessment of different markers of cardiovascular risk, good control of GFD compliance, and inclusion of both a larger retrospective group and a prospective study. We analyzed different risks including lipid metabolism, antioxidant status, inflammation, and nutrient deficiency. Our results show the complexity of arteriosclerotic risk and can be also used for planning future study.

## 5. Conclusions

CD is a lifelong disease; therefore, a proper therapeutic approach should include nutritional treatment with GFD, but it should also predict and prevent later complications and concomitant diseases. Together with GFD, the emphasize should be put on the long-term care and follow-up of the CD patients with respect to the compliance to the GFD, prevention of nutrient deficiencies, and early diagnosis of comorbidities.

In children with CD treated with GFD, decreased level of serum folic acid resulting from unbalanced diet, together with increased BMI, may predispose to atherosclerosis in the long run. On the contrary, it was shown that patients on GFD have normal lipid profile. However, due to the high degree of noncompliance to GFD, further research is needed to prove the impact of GFD on the risk factors of atherosclerosis in children. Presented data suggest the need of further studies to determine the need for metabolic cardiovascular risk screening in children with CD.

## Figures and Tables

**Table 1:** Clinical characteristics of patients enrolled in the study.

Parameter	Number of patients
Sex	
Females	164
Males	113
All	277
Place of living	
City	174
Village	76
nd	27
Marsh scale	
I^o^	5
II^o^	7
III^o^	233
nd	32
Clinical presentation of CD	
Classical	156
Atypical	82
Silent	12
nd	32
Comorbidities	
Autoimmune thyroiditis	4
Chronic autoimmune urticaria	2
Alopecia areata	1
Depression	4
Anxiety disorders	13
Epilepsy	4
Migraine	7
Selective IgA deficiency	20

nd: no data.

**Table 2:** IgA anti-TG2 results in the retrospective group (after 5 years of GFD; *n* = 189) and the prospective group (after 1 year of GFD; *n* = 61).

	Retrospective group (*n* = 189)	Prospective group (*n* = 61)
Positive IgA anti-TG2	61 (32.3%)	36 (59%)

**Table 3:** Correlation between mean *z*-score BMI and mean increase in *z*-score BMI (in the prospective group) with compliance to the GFD.

Variable	Noncompliance to GFD		Compliance to GFD		*p* value	95% CI	
	Mean	SD	Mean	SD		Min	Max
Mean *z*-score BMI (all patients)	-1.06	0.72	0.41	12.18	0.62	-7.32	4.40
Mean increase in *z*-score BMI (prospective group)	0.36	0.40	0.91	8.58	0.84	-5.98	4.89

**Table 4:** Analysis of the biochemical parameters in celiac patients and controls. Significant results are shown with bold font.

Parameter	All CD patients (*n* = 277)		Reference group (*n* = 95)		*p* value	95% CI	
	Mean	SD	Mean	SD		Min	Max
**TCH**	**154.2**	**29.2**	**166.8**	**30.5**	**0.0010**	**-20.1**	**-5.1**
**TG**	**84.7**	**42.1**	**74.0**	**32.2**	**0.0325**	**0.9**	**20.6**
**LDL-C**	**88.8**	**27.9**	**101.8**	**27.2**	**0.0003**	**-20.0**	**-6.1**
VLDL-C	15.91	7.20	14.42	5.91	0.0868	-0.22	3.21
HDL-C	49.78	14.01	50.59	12.31	0.6394	-4.20	2.58
TCH-HDL-C	3.367	1.306	3.440	0.912	0.6354	-0.374	0.229
LDL-C-HDL-C	1.999	1.110	2.141	0.817	0.2766	-0.400	0.115
**ApoA1**	**1.385**	**0.366**	**1.483**	**0.239**	**0.0083**	**-0.170**	**-0.025**
**ApoB**	**0.66**	**0.20**	**1.05**	**0.32**	**0.00001**	**-0.46**	**-0.31**
**A1-B**	**2.31**	**1.00**	**1.57**	**0.59**	**0.00001**	**0.55**	**0.93**
**B-A1**	**0.558**	**0.438**	**0.724**	**0.254**	**0.0010**	**-0.264**	**-0.068**

SD: standard deviation; TCH: total cholesterol; TG: triglycerides; A1: apolipoprotein A1; B: apolipoprotein B; apoE: apolipoprotein E; Lp(a): lipoprotein (a); LDL-C: low-density cholesterol; VLDL-C: very low-density cholesterol; HDL-C: high-density cholesterol; TC/HDL-C, LDL-C/HDL-C, A1-B, and B-A1: indicators of atherosclerosis.

**Table 5:** Analysis of the biochemical parameters in the prospective group: test I was obtained on the day of diagnosis, and test II was obtained 12 months after introducing the gluten-free diet. Significant results are shown with bold font.

Parameter	Test I		Test II		*p* value
	Mean	SD	Mean	SD	
TCH	156.78	32.86	161.94	28.76	1.000
TG	69.86	31.45	58.90	28.40	0.839
LDL-C	94.98	29.32	100.37	26.57	1.000
VLDL-C	13.00	4.90	11.61	5.27	1.000
HDL-C	48.73	16.28	49.90	13.51	1.000
TCH-HDL-C	3.60	1.55	3.53	1.41	1.000
LDL-C-HDL-C	2.28	1.39	2.26	1.22	1.000
ApoA1	1.32	0.44	1.35	0.38	1.000
ApoB	0.71	0.22	0.78	0.23	1.000
AI-B	2.05	1.01	1.91	0.84	1.000
B-A1	0.69	0.55	0.70	0.55	1.000
ApoE	14.88	4.08	14.08	3.26	1.000
Lp(a)	16.94	11.78	16.49	11.39	1.000
**TAS**	**1.30**	**0.34**	**1.44**	**0.29**	**0.039**
Homocysteine	9.89	8.26	8.38	2.12	1.000
**Folic acid**	**12.07**	**9.34**	**5.61**	**2.09**	**0.001**
Uric acid	4.97	1.47	4.72	0.99	1.000
**hsCRP**	**0.57**	**0.37**	**0.39**	**0.19**	**0.029**

SD: standard deviation; TCH: total cholesterol; TG: triglycerides; apoA1: apolipoprotein A1; APOB: apolipoprotein B; APOE: apolipoprotein E; Lp(a): lipoprotein (a); LDL-C: low-density cholesterol; VLDL-C: very low-density cholesterol; HDL-C: high-density cholesterol; TAS: total antioxidant status; TC/HDL-C, LDL-C/HDL-C, and A1/B: indicators of atherosclerosis; hsCRP: high-sensitivity CRP.

**Table 6:** Analysis of the biochemical parameters in CD patients in the retrospective group and healthy controls (reference group). Significant results are shown with bold font.

	CD patients (retrospective group, *n* = 210)		Reference group (*n* = 95)		*p* value	95% CI	
Parameter	Mean	SD	Mean	SD		Min	Max
**TCH**	**153.6**	**28.8**	**166.8**	**30.5**	**0.0005**	**-20.6**	**-5.8**
**Tg**	**85.1**	**42.1**	**74.0**	**32.2**	**0.0269**	**1.3**	**21.0**
**LDL-C**	**88.2**	**27.5**	**101.8**	**27.2**	**0.0001**	**-20.5**	**-6.7**
VLDL-C	15.98	7.21	14.42	5.91	0.0748	-0.16	3.28
HDL-C	49.73	14.05	50.59	12.31	0.6196	-4.26	2.55
TCH-HDL-C	3.362	1.312	3.440	0.912	0.6121	-0.381	0.225
LDL-C-HDL-C	1.991	1.113	2.141	0.817	0.2539	-0.409	0.108
**APOA1**	**1.383**	**0.367**	**1.483**	**0.239**	**0.0072**	**-0.173**	**-0.027**
**APOB**	**0.66**	**0.20**	**1.05**	**0.32**	**0.00001**	**-0.46**	**-0.32**
**AI-B**	**2.32**	**1.00**	**1.57**	**0.59**	**0.00001**	**0.56**	**0.94**
**B-AI**	**0.557**	**0.441**	**0.724**	**0.254**	**0.001**	**-0.266**	**-0.068**
**hsCRP**	**0.39**	**0.31**	**0.52**	**0.42**	**0.00001**	**0.00**	**0.00**

SD: standard deviation; TCH: total cholesterol; TG: triglycerides; APOA1: apolipoprotein A1; APOB: apolipoprotein B; APOE: apolipoprotein E; Lp(a): lipoprotein (a); LDL-C: low-density cholesterol; VLDL-C: very low-density cholesterol; HDL-C: high-density cholesterol; TAS: total antioxidant status; TC/HDL-C, LDL-C/HDL-C, and A1/B: indicators of atherosclerosis; hsCRP: high-sensitivity CRP.

## Data Availability

The data used to support the findings of this study are included within the article and are available from the corresponding author upon request.
